# Identification of Potential Key circRNAs in Aged Mice With Postoperative Delirium

**DOI:** 10.3389/fnmol.2022.836534

**Published:** 2022-04-14

**Authors:** Wei Ran, Ning Liang, Ruixue Yuan, Zhiqiao Wang, Jin Gao

**Affiliations:** Department of Anesthesiology, The First Affiliated Hospital of Chongqing Medical University, Chongqing, China

**Keywords:** circRNA, microarray, postoperative delirium, aging, cognitive

## Abstract

Postoperative delirium (POD) is a common postoperative complication in elderly patients and seriously affects postoperative recovery. The exact mechanism of POD is still unclear. Therefore, it is necessary to explore the mechanism of POD in transcriptional regulation. At present, circRNAs have been proven to play an important role in a variety of mental health and cognitive disorders, such as Alzheimer’s disease, depression and schizophrenia. To reveal the effect of circRNA on POD, we used microarray to analyze the differential expression profiles of circRNAs in the hippocampus of 12-month-old mice between the tibial fracture and control groups. A total of 1,4236 circRNAs were identified. Compared with the control group, there were 500 circRNAs with increased expression and 187 with decreased expression. The accuracy of the microarray data was further verified by qRT–PCR. Finally, GO enrichment and KEGG pathway analyses indicated that changes in axon orientation, ubiquitin-mediated proteolysis, glutamate synapses, the estrogen signaling pathway, the RAS signaling pathway and other systems may be important potential pathological mechanisms in the progression of POD. In particular, we found that the *HOMER1* gene and its transcript mmu_circRNA_26701 are specifically expressed in the glutamate synapse, which may provide new clues and intervention targets for the progression of this refractory disease.

## Introduction

Postoperative delirium (POD) is a common postoperative complication in elderly patients (Mahanna-Gabrielli et al., 2019), especially in heart and orthopedic surgery (Naeije and Pepersack, 2014; Quan et al., 2019; Eckenhoff et al., 2020; Majewski et al., 2020; Yang et al., 2020). POD can accelerate the trajectory of cognitive decline (Needham et al., 2017) and increase the mortality rate in the first year after surgery (Davis et al., 2017). It is associated with an increased risk of Alzheimer’s disease (AD) (Koster et al., 2012; Inouye et al., 2016; Yang et al., 2020), which brings new challenges to our aging society and represents a major burden on families and the health care system (Dziegielewski et al., 2021).

Previous studies have suggested that POD patients and AD patients have very similar pathogenic and pathophysiological mechanisms (Lu et al., 2019; Huang et al., 2020). However, the exact pathogenesis of POD is still unclear. This may be related to central nervous system inflammation, blood–brain barrier disruption, endoplasmic reticulum stress, mitochondrial damage, cholinergic system disorder, BDNF decline, amyloid beta accumulation and tau protein hyperphosphorylation (Wang et al., 2017; Netto et al., 2018; Ni et al., 2018; Ye et al., 2020). In recent years, many studies have found that abnormal circRNA expression is closely related to cognitive dysfunction observed in various mental health diseases, such as AD (Ma et al., 2020; Mehta et al., 2020). However, we cannot directly obtain the hippocampal tissue of POD patients in clinical practice, so it is important to explore the effect of circRNA on POD in animal models. At present, there are few studies investigating circRNAs and their functions are not fully understood. Our study attempts to provide a preliminary assessment for the potential role of circRNAs in the progression of POD.

In recent years, studies on the regulation of gene transcription in POD have increased. Gao et al. (2020) analyzed the differential expression profile of circRNAs in the blood of POD patients and found that circRNAs are involved in the regulation of development processes, cell adhesion and nervous system development and are enriched in the MAPK signaling pathway and RAS signaling pathway. Wu W. et al. (2021) analyzed the expression of circRNAs, miRNAs and mRNAs in the prefrontal cortex by NGS sequencing technology and believed that neuroinflammation was the main pathological mechanism of POD. Wu Y. Q. et al. (2021) used microarray and Cytoscape analysis to predict that the HUB genes Egfra and Prkacb regulate POD progression.

However, there are still few studies on the involvement of circRNAs in the pathogenesis of POD. We used microarray technology to analyze the differential expression profile of circRNA in the hippocampus of 12-month-old mice, which may provide new insights for research on the progress and intervention targets of circRNA on POD in aged mice.

## Materials and Methods

### Animals

This experiment was carried out in accordance with the Guidelines for the Care and Use of Experimental Animals and approved by the Animal Ethics Committee of the First Affiliated Hospital of Chongqing Medical University. Eighteen 12-month-old male C57BL/6J mice were purchased from the Animal Experimental Center of Chongqing Medical University (Chongqing, China), weighed 30–35 g, and were group-housed with standard rodent chow for 1 week (control, surgery; temperature 25°C, humidity 55%, 12-h light/dark cycles). All the animals were used for behavioral studies, and the survival rate following surgery was 100%. The success rate of modeling was 88.9% (one sample in the surgery group was excluded from the study due to modeling failure). The six samples in the microarray chip analysis were selected randomly from the successfully modeled control group and the experimental group. Another six samples were also randomly selected from the two groups for histological analysis. Investigators who performed surgery on the animals were not blinded to the treatment groups and collected samples, which were then analyzed by other investigators who were blinded to the specific treatment.

### Animal Model of Postoperative Delirium

Intramedullary nail fixation of tibial fracture on the left hind leg was performed under isoflurane anesthesia (2% isoflurane induction, 1.5% maintenance; Baxter International, Deerfield, IL, United States) and subcutaneous butylphenol analgesia (0.1 mg/kg, Jiangsu Hengrui Pharmaceutical Co., LTD., Lianyungang, China). Details were as follows: the temperature was maintained with a heating pad, all mice were fixed in the supine position after successful anesthesia, the left hind leg was disinfected with povidone iodine 3 times after skin preparation, and a longitudinal incision of approximately 1 cm was made in the middle and upper tibia of the left hind leg to fully expose the tibia. Osteotomy was performed in the middle part of the tibia, and then a 0.38 mm diameter sterile steel needle was inserted into the tibial medullary cavity. The end of the steel needle was cut at the level of the tibial plateau. After rinsing the incision, the wound was closed with 5-0 nylon sutures, the maintenance of anesthesia was stopped, and the mice were placed back into the cage to wake up naturally. All surgeries were completed within 15 min. To eliminate the effects of anesthesia and analgesia on cognition, mice in the control group received the same dose of anesthesia and analgesia.

### Fear Conditioning Test

The fear conditioning test (FCT) is designed to identify how animals learn and remember unpleasant experiences related to the environment. Sound and light are commonly used as conditioned stimulus (CS), while aversive stimuli (such as electric shocks to the feet) are used as unconditioned stimuli (US). These two types of stimulation appeared in pairs during the test. The animals were able to make CS-US connections between the shock and the environment in addition to the connection between the sound and the shock. Our experimental design was slightly modified on the basis of previous studies (Wei et al., 2017; Li et al., 2019; Xiang et al., 2019). The mice were put into the test chamber on the preoperative day and 30 min before surgery (9:00 am). After 3 min of free exploration, the mice received three pairs of sound stimulation (5,000 Hz, 80 dB and 30 s) and electric shock stimulation (0.8 mA, 2 s). The sound should precede the shock, with an interval of 58 s between the two stimuli. The Panlab fear condition device was used to record the freezing time of mice in both contextual and cued tests, and all data were analyzed by PACKWIN v2.0.05 (Šmidák et al., 2016; Tishkina et al., 2016).

Contextual and cued tests were performed at 09:00 am on Day 3 after surgery. In the contextual test, mice were allowed to explore without the stimulation of sound and electric shock for 5 min; 2 h later, mice were placed in a different environment from the preoperative training environment (internal color change). After a 3-min exploration, the mice were given the same sound stimulation (5,000 Hz, 80 dB and 30 s) but no electric shock. The interval between the two stimulations was still 58 s. The box was wiped with alcohol to ensure that the next animal will not be affected by the smell of the previous mice; all tests were performed with the same equipment and analyzed by the same software.

### Open Field Test

The open field test (OFT) was used to evaluate the anxiety and activity levels of the experimental animals. Mice were placed in the same corner of the experimental box (45 cm × 45 cm × 60 cm, length × width × height) as in previous studies (Akillioglu et al., 2012; van Zyl et al., 2016; Zeldetz et al., 2018). During the 3-min test, the activity data of mice (the residence time and distance of mice in the central and marginal zone) were recorded by a digital camera, and the data were analyzed by Panlab SMART 3.0.

### Immunochemistry Analysis

The hippocampal tissue was fixed in 4% formaldehyde, followed by regular dehydration, transparent paraffin embedding, sectioning, and dewaxing. After hematoxylin & eosin HE staining, further dehydration, transparency, and sealing, the slides were analyzed under a light microscope (100×; Leica DM2000, Germany).

### RNA Extraction

The hippocampal tissues of the surgery and control group mice were harvested on Day 3 after surgery and stored at −80°C until use. Total RNA was extracted from the hippocampal samples of both groups using TRIzol reagent (Invitrogen, Carlsbad, CA, United States) according to the manufacturer’s instructions. The OD260/OD280 ratio of each sample ranged from 1.95 to 2.07, and the OD260/OD230 ratios were greater than 1.9, indicating good RNA quality (Supplementary Figure 2).

### Quantitative Real-Time PCR

qRT–PCR was performed using the TAKARA PrimeScript RT reagent Kit TRR037A reverse transcription polymerase chain reaction system (TAKARA, Japan) according to the manufacturer’s instructions. The specific primer pairs used in this study are listed in Table 1. Relative gene expression was calculated using the 2^–ΔΔCt^ method, and β-actin mRNA was used as a normalizer gene. All experiments were performed in triplicate.

**TABLE 1:** The forward and reverse primers for qRT-PCR.

Gene	Primers	Sequence (5′–3′)
mmu_circRNA_32003	Forward primer	TCATCAGATTTGCCAGGTTTG
	Reverse primer	GCCCAGTAACACTTGTAACTCAGA
mmu_circRNA_016934	Forward primer	ATGCCAACAAGCACCTAGTCC
	Reverse primer	GGGAAGCAACTTGGAAGAATG
mmu_circRNA_32141	Forward primer	TGACAAGGGTCCGAACAGTG
	Reverse primer	TATCCTGGTGACAGCTATCTTCC
mmu_circRNA_29678	Forward primer	TATCCTGGTGACAGCTATCTTCC
	Reverse primer	GGATGAGTCTTGGTGGAGAGTTC
mmu_circRNA_22546	Forward primer	GGATGAGTCTTGGTGGAGAGTTC
	Reverse primer	GTTGTCGGAGTTGGAGAAGC
mmu_circRNA_29619	Forward primer	TTGTGTCTGTGATCTATGCCTGTG
	Reverse primer	CCGAGACGCAGTAGTAACAATCC
mmu_circRNA_29301	Forward primer	ACACCAGCCTTAAAGTACCAACAG
	Reverse primer	CGGCTACTCCCAATCTTCCTC
mmu_circRNA_009489	Forward primer	CGGCTACTCCCAATCTTCCTC
	Reverse primer	TGCATGGTCATAGTCTGAGGG
mmu_circRNA_40568	Forward primer	TGCATGGTCATAGTCTGAGGG
	Reverse primer	GCTGCTTGATTCGCAACTTC
mmu_circRNA_31992	Forward primer	GCTGCTTGATTCGCAACTTC
	Reverse primer	CATAGCTTGTTGTCTTCAGCACTG
mmu-circRNA_26701	Forward primer	GCATTGCCATTTCCACATAGG
	Reverse primer	GTGCTGAAGATAGGTTGCTCCC
Homer1	Forward primer	TTCTCCTCTGAGCATCATCTTTC
	Reverse primer	TCGTCTGTCCCATTGATACTTTC
β-actin	Forward primer	GTGGCCGAGGACTTTGATTG
	Reverse primer	CCTGTAACAACGCATCTCATATT

### Microarray

We randomly selected 3 pairs of hippocampal samples from the surgery group and the control group for microarray analysis. Total RNA from each sample was quantified using a NanoDrop ND-1000. Sample labeling and array hybridization were performed according to the manufacturer’s protocol (Arraystar Mouse circular RNA Mcrioarray V2.0). Briefly, total RNA was digested with RNase R (Epicenter, Inc.) to remove linear RNAs and enrich circular RNAs. Then, the enriched circular RNAs were amplified and transcribed into fluorescent cRNA utilizing a random priming method (Arraystar Super RNA Labeling Kit; Arraystar). The labeled cRNAs were purified by an RNeasy Mini Kit (Qiagen). The concentration and specific activity of the labeled cRNAs (pmol Cy3/μg cRNA) were measured by a NanoDrop ND-1000. One microgram of each labeled cRNA was fragmented by adding 5 μl 10 × Blocking Agent and 1 μl of 25 × Fragmentation Buffer and then heated at 60°C for 30 min. Finally, 25 μl 2 × Hybridization buffer was added to dilute the labeled cRNA. Fifty microliters of hybridization solution was dispensed into the gasket slide and assembled onto the circRNA expression microarray slide. The slides were incubated for 17 h at 65°C in an Agilent Hybridization Oven. The hybridized arrays were washed, fixed and scanned using the Agilent Scanner G2505C.

### Data Analysis

The collected array images were analyzed by Agilent feature extraction software (version 11.0.1.1). Quantile normalization and subsequent data processing were performed using the R software limma package. After quantile normalization of the raw data, circRNAs for which 2 out of two samples had flags in Present or Marginal (“All Targets Value”) were chosen for further data analysis. Fold change filtering was employed for identification of differentially expressed circRNA between the two groups. Gene ontology (GO) and KEGG pathway enrichment analyses were conducted through the standard computation method. Gene ontologies are organized into a hierarchy structure of annotation terms to promote analysis and interpretation at different levels. The top-level ontologies are biological process, cellular component, and molecular function. Thus, GO database analysis was employed to reflect genetic regulatory systems based on the differentially expressed circRNAs in the biological process, cellular component and molecular function classification. KEGG database analysis was applied to identify the potential key pathways related to the differentially expressed genes. When comparing the contour differences between the two groups, the “fold range” (i.e., the ratio of the group averages) for each circRNA between the groups was calculated. The statistical significance of the difference was conveniently estimated by *t*-test. The circRNAs with fold changes greater than or equal to 1.3 and a *p*-value ≤ 0.05 were chosen as the significant differential expression. The microarray analysis was performed by KangChen Biotech (Shanghai, China).

### Statistical Analysis

Statistical analysis was performed by GraphPad Prism 8.3.0 (GraphPad, La Jolla, CA, United States). All data are expressed as the mean ± SEM. The difference between the two groups was tested by double-tailed Student’s *t*-test, and *P* < 0.05 was considered statistically significant.

## Results

### Tibial Fracture Induced Cognitive Impairment in Aged Mice

**FIGURE 1:**
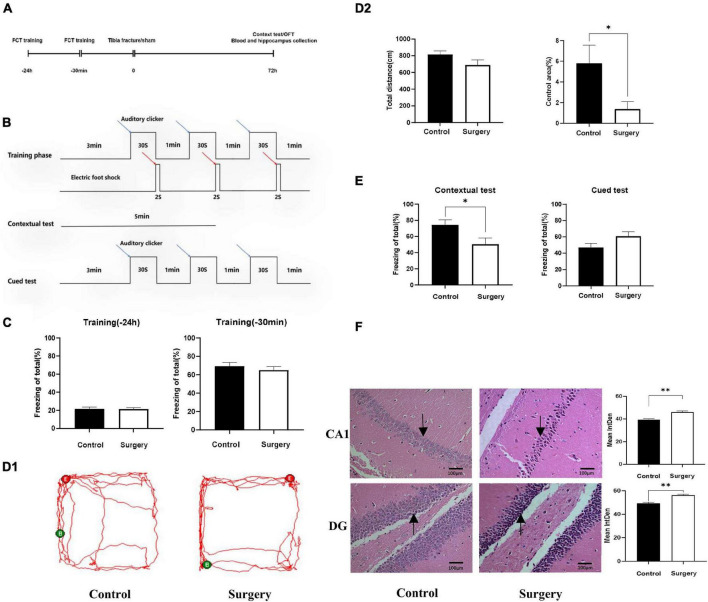
Cognitive disorder induced by tibial fracture in aged mice. **(A)** Experimental design. **(B)** FCT experimental design. **(C)** Preoperative FCT training. **(D1)** Mice trajectory, Green (B) and Red (E) indicate starting and ending point. **(D2)** Travel distance and proportion in central area. **(E)** Contextual test and cued test. **(F)** HE staining, black arrow: neurons contracted and nerve fiber tangles. Data are presented as Means ± SEM, analyzed by Student’ s *t*-test. CA1, DG means the CA1, DG region of hippocampus. IntDen means Integrated Density, analyzed by Image J. *P < 0.05 (*N* = 9). *P < 0.05, **P < 0.01.

Experimental procedures and the design of the fear conditioning test (FCT) are shown in Figures 1A,B. There was no significant difference in FCT freezing time between the two groups during training 1 day and 30 min before surgery (*P* > 0.05), as shown in Figure 1C. In the open field test (OFT), which was conducted on the third day after surgery, there was no significant difference in walking distance between the two groups (*P* > 0.05), but mice in the surgery group spent significantly less time in the central area of the arena (*P* < 0.05), as shown in Figures 1D1,D2. On the third day after surgery, the freezing time of mice in the surgery group was significantly decreased (*P* < 0.05), while there was no significant difference in the freezing time of mice in the two groups during the cued test (*P* > 0.05), as shown in Figure 1E. HE staining of the hippocampus of the two groups of mice indicated that in the surgery group, neurons in the hippocampal CA1 and DG areas were pyknotic and formed neurofibrillary tangles, as shown in Figure 1F. These data suggest that mice developed cognitive impairment on Day 3 after intramedullary nailing for tibial fractures.

### Differential Expression Profile of circRNA in the Mouse Hippocampus of the Postoperative Delirium and Control Groups

**FIGURE 2:**
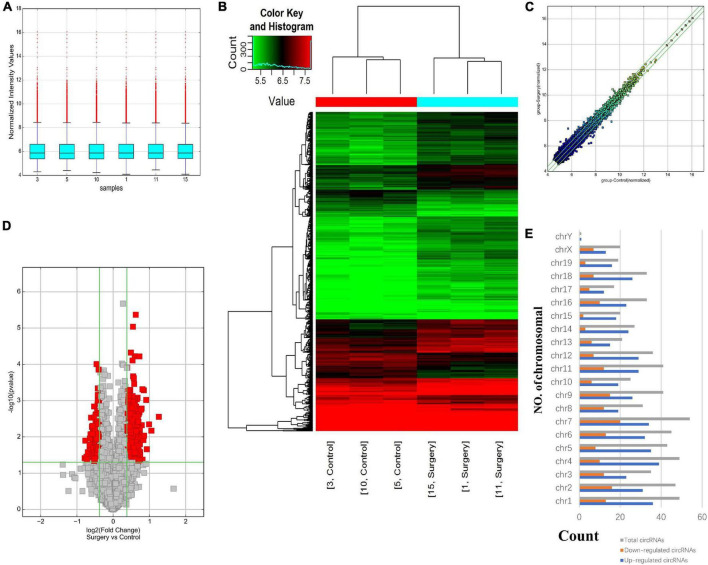
Differentially expressed profile of circRNAs and characterization between the two groups. **(A)** Box plots showing the distribution of circRNAs between the hippocampus samples, abscissa reports sample name and ordinate reports the normalized intensity values, the upper and lower sides of the rectangular box mean minimum and maximum values, the upper and lower lines of the error bars mean interquartile range, the line inside the rectangular box means median value. **(B)** Hierarchical clustering plot showing the differentially expressed circRNA profiles in the six samples. “Red” represents the higher expression, while “green” represents the lower expression level. **(C)** The difference expression of circRNA is shown by a scatter plot between the two groups. **(D)** Volcano plots visualizing the distinguishable circRNA expression, the abscissa represents significance, the ordinate represents multiple of expression difference. **(E)** Chromosomal distributions of circRNAs in the two groups.

Using a mouse circRNA microarray, we created a box plot to show the intensity distribution. We observed that the distribution of normalized intensity values was similar across the test sample (Figure 2A). The results of systematic cluster analysis showed that the expression levels of circRNA in the 6 samples were different, indicating that the expression patterns of circRNA in the surgery group were different than the control mice (Figures 2B,C). Significant differences between the two groups are shown by volcanic plots (fold change ≤ −1.3 and ≥1.3, *P*-value ≤ 0.0 5) (Figure 2D). In addition, the distribution of differentially expressed circRNAs on chromosomes showed that most circRNAs were transcribed from CHR1, CHR2, CHR4, and CHR7, and few were transcribed from CHR15, CHR17, CHR19, and chrY (Figure 2E). These data suggest that the expression pattern of circRNA in the hippocampus of mice undergoing surgery is different from that of the control group. Microarray data also revealed 687 differentially expressed circRNAs, of which 500 were upregulated and 187 were downregulated. Among them, mmu_circRNA_32003 had the highest expression level, and mmu_circRNA_29619 had the lowest expression level. The top 10 upregulated and downregulated circRNAs were reported (Table 2).

**TABLE 2:** Biological information for the top 10 upregulated and downregulated circRNAs.

CircRNA ID	Fold change	*P*-value	Chromosome	circRNA type	Best transcript	Gene symbol
Up-regulated						
mmu_circRNA_32003	2.3956284	0.002803458	chr18	Exonic	NM_008602	Pias2
mmu_circRNA_016934	2.0784582	0.006757496	chr7	Sense overlapping	ENSMUST00000178266	Gm22632
mmu_circRNA_32616	1.9856959	0.004529479	chr19	Exonic	NM_015748	Slit1
mmu_circRNA_19057	1.9029061	0.02189749	chr12	Sense overlapping	NM_030723	Pum2
mmu_circRNA_32141	1.8831334	0.000961373	chr19	Exonic	NM_028999	Ppp6r3
mmu_circRNA_40596	1.8078959	0.009315359	chr6	Exonic	NM_011666	Uba3
mmu_circRNA_29678	1.7980695	0.000519849	chr16	Exonic	NM_172440	Stxbp5l
mmu_circRNA_22546	1.7848188	0.012217361	chr10	Exonic	NM_177368	Tmtc2
mmu_circRNA_43782	1.755117	0.042530691	chr9	Intronic	ENSMUST00000058796	Kdm4d
mmu_circRNA_29342	1.751172	0.010890393	chr16	Exonic	NM_139232	Fgd4
Down-regulated						
mmu_circRNA_29619	−1.7246528	0.038610999	chr16	Exonic	NM_001012765	Adcy5
mmu_circRNA_39436	−1.7036053	0.012698343	chr5	Exonic	NM_021311	Piwil1
mmu_circRNA_011181	−1.6684252	0.035218869	chr8	Exonic	NR_037865	Ankrd11
mmu_circRNA_29301	−1.6489009	0.041686479	chr16	Exonic	NM_008565	Mcm4
mmu_circRNA_42016	−1.6245053	0.026518293	chr7	Exonic	NM_001286689	Xndc1
mmu_circRNA_27381	−1.5951465	0.045779044	chr14	Exonic	NM_001081251	Pbrm1
mmu_circRNA_009489	−1.594478	0.047084917	chr6	Intronic	ENSMUST00000172974	Calu
mmu_circRNA_23144	−1.5695885	0.018989182	chr11	Sense overlapping	NM_001100394	4930505A04Rik
mmu_circRNA_40568	−1.5650436	0.016436792	chr6	Exonic	NM_001081146	Prickle2
mmu_circRNA_31992	−1.5592829	0.011072928	chr18	Sense overlapping	NM_145356	Zbtb7c

### Bioinformatics Analysis of the Predicted Network Genes for Differentially Expressed circRNAs

**FIGURE 3:**
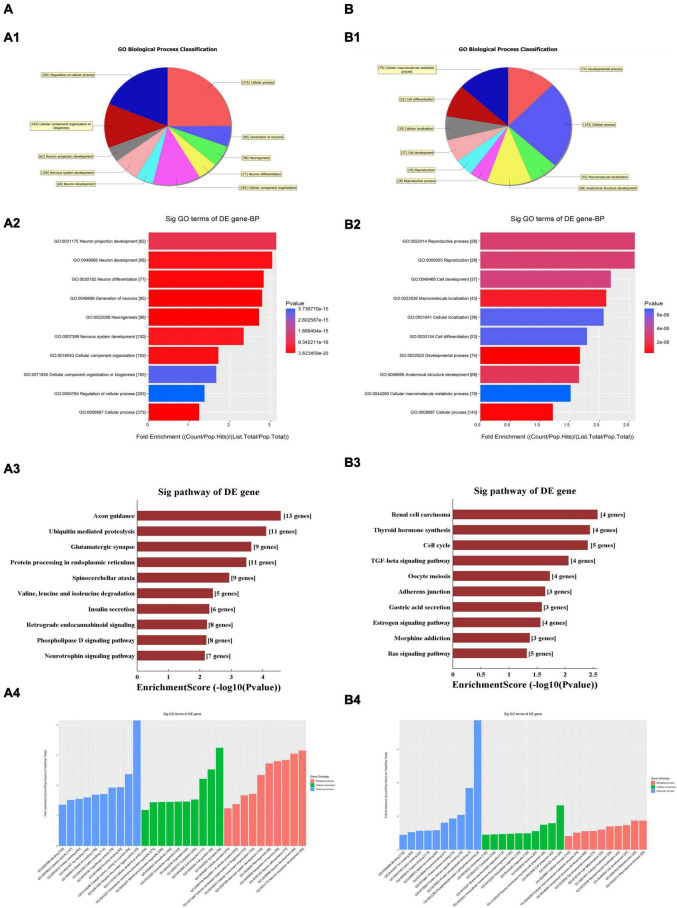
Gene Ontology (GO) and Kyoto Encyclopedia of Genes and Genomes (KEGG) analyses for upregulated and downregulated **(A,B)** circRNAs. **(A1,B1)** Classification of the GO analysis predicted biological processes. **(A2,B2)** Top 10 significantly enriched genes based on their fold enrichment scores. **(A3,B3)** KEGG pathway analysis showing the top 10 significantly enriched pathways and their scores. **(A4,B4)** GO analysis predictions in cellular component and molecular function. GO gene ontology; Sig, significantly; BP, biological processes. Selection counts, count of the genes’ entities directly associated with the listed pathway ID; Selection Size, the total number of the genes’ entities.

The GO and KEGG pathway analyses were performed for functional enrichment analysis of these differentially expressed circRNAs. In the GO analysis, the upregulated biological process was mainly involved in neuron projection development, as well as neuron generation, differentiation and development, while the downregulated biological process mainly included cell process and cellular macromolecule metabolism. In the GO molecular function analysis, the most significantly upregulated and downregulated GO functional items were ubiquitin-like protein ligase activity and phosphatidylinositol-3-phosphate binding, respectively. In the GO cell component analysis, the most significantly upregulated and downregulated GO functional items were synaptic function and axon function, respectively. In KEGG pathway analysis, we showed 10 upregulated pathways and 10 downregulated pathways with the most significant differences, among which upregulated pathways mainly included axonal orientation, ubiquitin-mediated proteolysis and glutamate synapses, and downregulated pathways mainly included the TGF-β signaling pathway, estrogen signaling pathway and RAS signaling pathway. The complete information of all GO functional enrichment and KEGG pathways is shown in Figure 3.

### Predicted miRNAs and Differentially Expressed Genes in the Pathway Map

**FIGURE 4:**
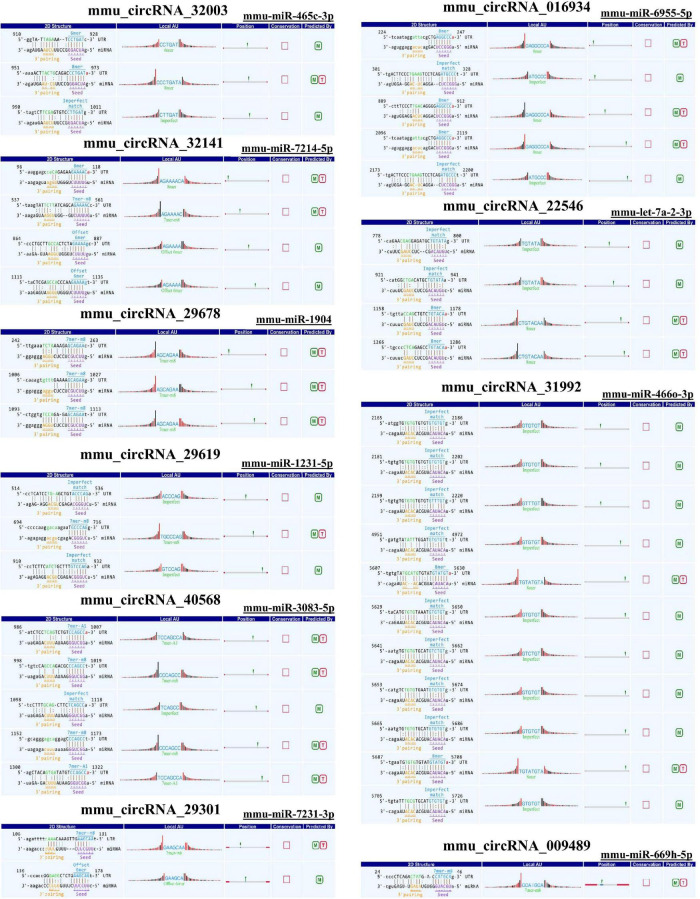
Sequence pairing predictions for circRNAs and miRNAs. Seed sequence matching predicted the direct interaction of the abovementioned circRNAs with their related miRNAs.

**FIGURE 5:**
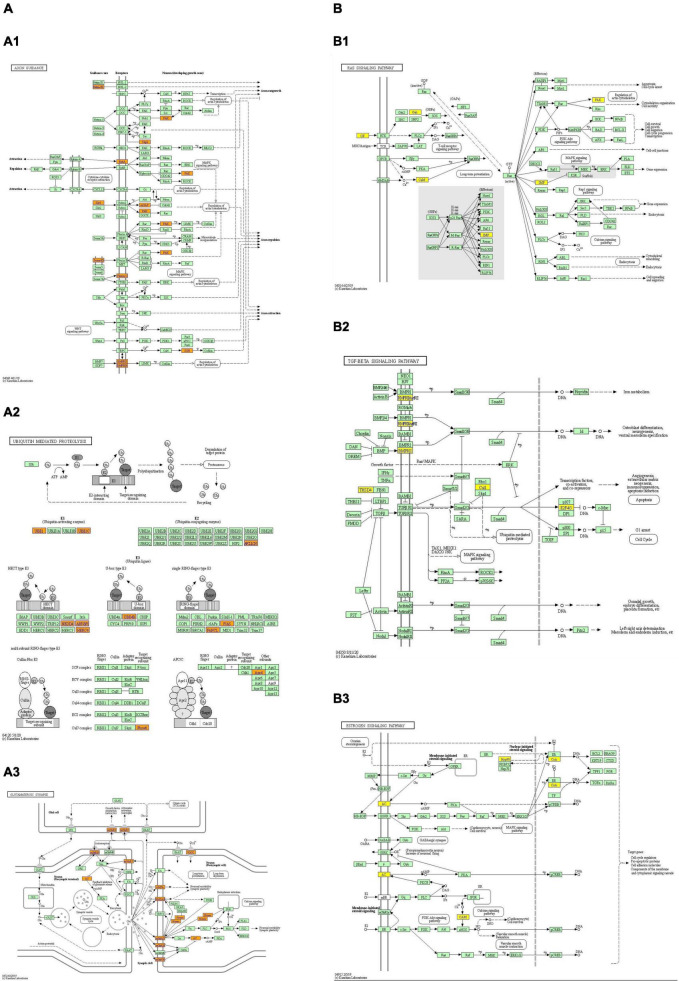
The top 3 upregulated and downregulated **(A,B)** KEGG pathway map. Orange marked nodes are associated with up-regulated or only whole dataset genes, yellow marked nodes are associated with down-regulated genes, green nodes have no significance. Up-regulated pathways: **(A1)** axon guidance; **(A2)** ubiquitin mediated proteolysis; **(A3)** glutamatergic synapses. Down-regulated pathways: **(B1)** RAS signal pathway; **(B2)** TGF- β signal pathway; **(B3)** estrogen signal pathway.

As there are specific binding sites of miRNAs in the circRNA sequence, circRNAs can interact with miRNAs by miRNA response elements. The five miRNAs with the highest mirSVR scores among the differentially expressed circRNAs, including the top five upregulated and downregulated circRNAs, are shown in Table 3 and Figure 4. Pathway analysis is a functional analysis mapping genes to KEGG pathways. The *p*-value (EASE score, Fisher *P*-value or hypergeometric *p*-value) denotes the significance of the pathway correlated to the conditions. The lower the *p*-value is, the more significant the pathway. We provided all differentially expressed genes in the top 3 upregulated and downregulated pathways (Figure 5). The top 10 upregulated and downregulated signal pathway regulation information are shown in Table 4.

**TABLE 3:** Predicted miRNA response elements of the confirmed circRNAs.

CircRNA ID	Predicted miRNA response elements (MREs)				
	MRE1	MRE2	MRE3	MRE4	MRE5
**Up-regulated**					
mmu_circRNA_32003	mmu-miR-465c-3p	mmu-miR-465b-3p	mmu-miR-465a-3p	mmu-miR-7007-5p	mmu-miR-499-3p
mmu_circRNA_016934	mmu-miR-6955-5p	mmu-miR-7665-5p	mmu-miR-6379	mmu-miR-6988-3p	mmu-miR-5621-5p
mmu_circRNA_32141	mmu-miR-7214-5p	mmu-miR-6946-3p	mmu-miR-3071-5p	mmu-miR-26a-2-3p	mmu-miR-7021-3p
mmu_circRNA_29678	mmu-miR-1904	mmu-miR-6344	mmu-miR-6946-3p	mmu-miR-29b-2-5p	mmu-miR-21b
mmu_circRNA_22546	mmu-let-7a-2-3p	mmu-miR-677-3p	mmu-miR-3083-5p	mmu-miR-222-5p	mmu-miR-1a-3p
**Down-regulated**					
mmu_circRNA_29619	mmu-miR-1231-5p	mmu-miR-449a-5p	mmu-miR-6905-5p	mmu-miR-493-3p	mmu-miR-7034-3p
mmu_circRNA_29301	mmu-miR-7231-3p	mmu-miR-6933-3p	mmu-miR-1264-3p	mmu-miR-6999-3p	mmu-miR-6946-3p
mmu_circRNA_009489	mmu-miR-669h-5p	mmu-miR-7688-3p	mmu-miR-7652-3p	mmu-miR-7091-3p	mmu-miR-1943-3p
mmu_circRNA_40568	mmu-miR-3083-5p	mmu-miR-1903	mmu-miR-7028-3p	mmu-miR-6357	mmu-miR-6342
mmu_circRNA_31992	mmu-miR-466o-3p	mmu-miR-466f-3p	mmu-miR-466q	mmu-miR-5110	mmu-miR-466m-3p

**TABLE 4:** Predicted differentially expressed genes in the top ten up-regulated and down-regulated pathways.

PathwayID	Definition	P-value	Enrichment_Score	GeneRatio	Genes
**Up-regulated**					
mmu04360	Axon guidance	0.00002702891	4.568171	0.079268	BMPR1B//BMPR2//EPHA3//EPHA6//EPHA7//NTNG2//PAK7//PLXNC1//PTPN11//SEMA4A//SLIT1//SRGAP1//SSH2
mmu04120	Ubiquitin mediated proteolysis	0.00007801933	4.107798	0.067073	ANAPC4//BIRC6//FANCL//FBXW8//HERC4//HUWE1//NEDD4L//PIAS2//UBA3//UBA6//UBE4B
mmu04724	Glutamatergic synapse	0.000232778	3.633057	0.054878	ADCY5//ADCY6//CACNA1C//GRIA1//GRIN3A//GRM3//GRM5//GRM7//HOMER1
mmu04141	Protein processing in endoplasmic reticulum	0.00033252	3.478183	0.067073	ATF6//ATXN3//CANX//MAPK10//MBTPS2//SEC23B//SEC24A//SEC24D//SEC61A2//UBE4B//UBQLN1
mmu05017	Spinocerebellar ataxia	0.001175436	2.929801	0.054878	ATXN3//GRIA1//GRIN3A//MAPK10//PIK3C3//PSMA6//PSMD8//PUM2//RELN
mmu00280	Valine, leucine and isoleucine degradation	0.003909513	2.407877	0.030488	ABAT//BCAT2//MCCC1//PCCA//PCCB
mmu04911	Insulin secretion	0.005051402	2.296588	0.036585	ADCY5//ADCY6//CACNA1C//CREB3L2//KCNN2//KCNN3
mmu04723	Retrograde endocannabinoid signaling	0.006058934	2.217604	0.04878	ADCY5//ADCY6//CACNA1C//CNR1//GRIA1//GRM5//MAPK10//NDUFA9
mmu04072	Phospholipase D signaling pathway	0.006304622	2.200341	0.04878	ADCY5//ADCY6//DGKB//GRM3//GRM5//GRM7//PIP5K1B//PTPN11
mmu04722	Neurotrophin signaling pathway	0.007022585	2.153503	0.042683	FOXO3//KIDINS220//MAPK10//NTRK3//PDPK1//PTPN11//SORT1
**Down-regulated**					
mmu05211	Renal cell carcinoma	0.0026713	2.573277	0.052632	EPAS1//GAB1//HIF1A//PAK1
mmu04918	Thyroid hormone synthesis	0.003631412	2.439925	0.052632	ADCY5//CANX//GSR//HSP90B1
mmu04110	Cell cycle	0.003972019	2.400989	0.065789	CUL1//DBF4//E2F5//MCM4//YWHAE
mmu04350	TGF-beta signaling pathway	0.008782051	2.056404	0.052632	BMPR2//CUL1//E2F5//THSD4
mmu04114	Oocyte meiosis	0.01879689	1.725914	0.052632	ADCY5//CALM3//CUL1//YWHAE
mmu04520	Adherens junction	0.02274874	1.643043	0.039474	NLK//PARD3//PTPRF
mmu04971	Gastric acid secretion	0.02623478	1.581123	0.039474	ADCY5//CALM3//KCNQ1
mmu04915	Estrogen signaling pathway	0.02763593	1.558526	0.052632	ADCY5//CALM3//HSP90B1//NCOA1
mmu05032	Morphine addiction	0.04286854	1.367861	0.039474	ADCY5//PDE2A//PDE8B
mmu04014	Ras signaling pathway	0.04831079	1.315956	0.065789	BRAP//CALM3//GAB1//IGF2//PAK1

### Amplification and Identification of Differentially Expressed circRNAs

**FIGURE 6:**
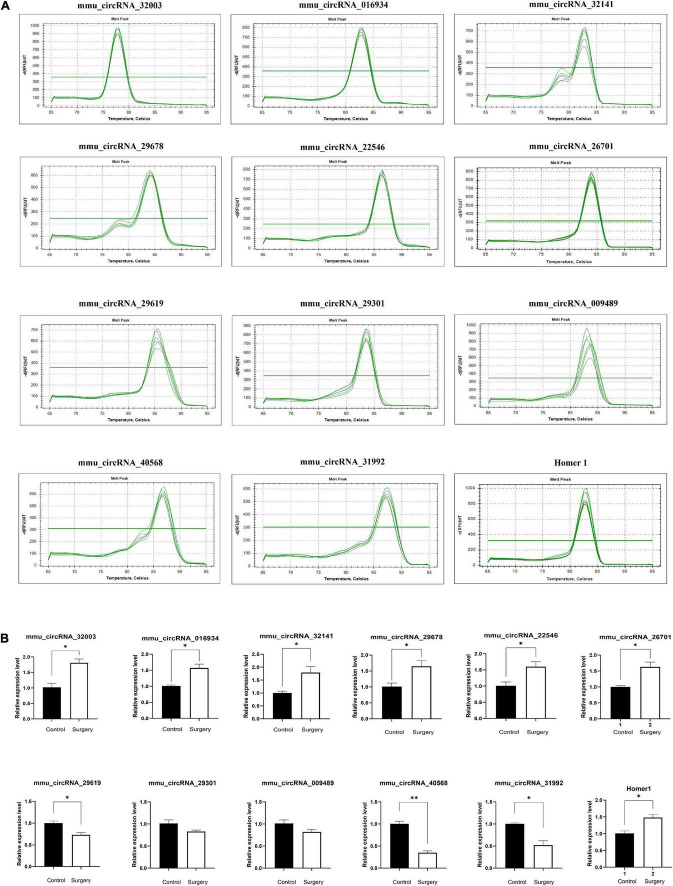
Amplification and identification of differentially expressed circRNAs. **(A)** The melt curves of the identified distinguishable expressed circRNAs. **(B)** qRT-PCR showing the expression levels of circRNAs between the two groups. Homer1 mRNA was used to detect the expression level of Homer1 gene, while mmu_circRNA_26701 was a circular RNA transcribed from Homer1 gene. Data are presented as means ± SEM, analyzed by Student’s *t*-test. *P < 0.05, ^**^P < 0.01. (*N* = 6).

To further verify the accuracy of the results, we randomly selected 5 pairs of differentially expressed circRNAs for further qRT–PCR analysis and identified the PCR results through dissolution curve analysis. Thus, the primers can specifically amplify the post-splicing sites of circRNA (Figure 6A). Compared with the control group, mmu_circRNA_32003, mmu_circRNA_016934, mmu_circRNA_32141, mmu_circRNA_29678, and mmu_circRNA_22546 expression increased, and the expression levels of mmu_circRNA_29619, mmu_circRNA_40568 and mmu_circRNA_31992 decreased (Figure 6B). The results of qRT–PCR analysis is consistent with those of our microarray analysis, which confirms the reliability of the microarray data. Finally, we found that Circ_Homer1 was the specifically altered circRNA in the regulation of all differential signaling pathways in the synaptic regulation of glutamate release. We confirmed by qRT–PCR that Circ_Homer1 and Homer1 expression levels increased in the hippocampal tissues of the surgery group (Figures 6A,B).

## Discussion

In this study, we selected 12-month-old mice as the research subjects. The POD model was established by intramedullary nail fixation of tibial fractures as reported in previous studies (Feng et al., 2017; Xiong et al., 2018), and, for the first time, the HE staining index of hippocampal neurons was used to evaluate modeling success. Subsequently, 3 pairs of POD mice and normal control mice were randomly analyzed by microarray, and 14,236 differentially expressed circRNAs were identified in the hippocampus. A total of 687 differentially expressed circRNAs were identified, including 500 upregulated genes and 187 downregulated genes. GO and KEGG pathway enrichment analyses of the differentially expressed genes indicated that Axon guidance, Ubiquitin proteolysis and Glutamatergic synapse pathways were upregulated, while RAS, TGF-β and Estrogen signaling pathways were downregulated. We also noticed that HOMER1 gene was specifically increased in Glutamate synaptic pathway. Further data analysis found that mmu_circRNA_26701 expression level increased significantly and was transcribed from the HOMER1 gene. Subsequently, we verified the high expression of mmu_circRNA_26701 and HOMER1 by qRT–PCR. Therefore, our study identifies novel circRNAs that may be involved in the regulation of POD progression, which may provide new targets for the treatment and intervention of this refractory disease.

Although similar studies have been conducted before (Wu Y. Q. et al., 2021), we still have some innovations in this study. First, we chose 12-month-old mice as subjects to avoid the potential influence of age (which may have resulted in cognitive deficits before the experiment) (Benice et al., 2006). This may provide new insights into the pathogenesis of POD. Second, in the FCT test, we trained mice with sound (5,000 Hz, 80 dB and 30 s) and electric stimulation (0.8 mA, 2 s). These methods have been well validated in previous studies (Feng et al., 2017; Wei et al., 2017). Third, in POD modeling, we used intramedullary nail fixation instead of intramedullary nail implantation of tibial fracture, and how changing the modeling method affects the outcome is unknown. Fourth, we stained the hippocampal tissues of mice with HE and found neuronal contraction and neurofibrillary tangles in the DG and CA1 areas of hippocampal tissue after surgery, which was consistent with the conclusion of Chen et al. (2021) based on cognitive dysfunction after fine particulate matter (PM2.5) exposure, which provides new evidence for POD modeling. Fifth, Wu Y. Q. et al. (2021) analyzed the interaction between proteins mainly through microarray chip technology and Cytoscape technology and predicted that the HUB genes were regulated by circRNAs. Our study focused more on the effects of circRNAs on synaptic function. Sixth, they identified only a small number of differentially expressed genes. Therefore, our study provides new insights into animal age selection, modeling methods, modeling success criteria, and experimental purposes. These rigorous and unique choices provide strong support for a new understanding and reliability of conclusions on the pathogenesis of postoperative delirium, and they may expand our traditional understanding of the pathogenesis of POD.

As indicated in GO and KEGG analyses, the target genes of these circRNAs are involved in neural development and differentiation (GO: 0031175) (GO: 0048666) (GO: 0030182) (GO: 0007399) and synapses (GO: 0045202), and KEGG pathway analysis showed that the upregulated pathways mainly included axonal orientation, ubiquitin-mediated proteolysis, glutamate-energy synapses, phospholipase D signaling pathway, and neurotrophin signaling pathway. Downregulated pathways mainly include the TGF-β signaling pathway, estrogen signaling pathway, RAS signaling pathway, etc. KEGG analysis has enriched the regulatory network of multiple signaling pathways in POD progression, and many signaling pathways have been fully validated in AD models. For example, axon-guiding molecules play a role in the occurrence and development of AD by participating in different mechanisms (Zhang et al., 2021). Dysregulation of UPP (ubiquitin–proteasome pathway) protein degradation contributes to cognitive impairment in aging and AD (Hegde et al., 2019), and in particular, the local regulation of ligases in neurons may be important (Hegde and Upadhya, 2007). Similarly, age-related cognitive decline is associated with synaptic loss and/or changes in synaptic proteins (Pereira et al., 2014). While estrogen signaling pathways have been reported to play an important role in cognitive protection (Engler-Chiurazzi et al., 2017; Hwang et al., 2020), TGF-β and RAS signaling pathways are involved in the regulation of inflammation and apoptosis, leading to cognitive impairment (Kirouac et al., 2017; Kashima and Hata, 2018). It has been reported that the parent gene ITSN1 of Hsa_CircRNA_061570 impairs synaptic plasticity and learning and memory function of AD by activating RAS-JNK signaling pathway (Yarza et al., 2015). Therefore, our GO and KEGG analyses obtained more differentially expressed genes, which expanded the signaling pathways previously dominated by inflammation and apoptosis, so that more target genes were enriched in different signaling pathways, especially in the regulation of nervous system development and synaptic function.

At present, glutamate *trans*-synaptic transmission is considered to be closely related to the development and treatment of various mental health and cognitive diseases. Glutamate signals are processed by a variety of receptors, mainly including metabolic glutamate receptors (MGluRs), which regulate downstream Ca^2+^ signals by interacting with the scaffold protein Homer 1 (Conway, 2020; Gigg et al., 2020). Wagner et al. (2013) demonstrated that acute stress leads to cognitive deficits mediated by mGluR5/Homer 1 signaling in the hippocampus. Homer 1, a recognized regulator of synaptic plasticity and neuronal excitability, has been implicated in a plethora of mental health and cognitive disorders, including schizophrenia and depression (Hu et al., 2010; Wagner et al., 2013; Serchov et al., 2020). Zimmerman et al. (2020) demonstrated that CircHomer1a is involved in the regulation of frontal cortex development, which is necessary for cognitive flexibility, and it strongly alters the expression of many mRNA isomers of genes associated with synaptic function and psychiatric disorders. In our study, high expression of Homer1 in the differentially expressed genes of glutamate synapses was also found. Further tracing revealed that mmu-circrNA_26701 was transcribed from it. Then, qRT–PCR was used to verify the high expression of Homer1 and circRNA_26701, which was consistent with the expected results. However, until now, POD-related circRNAs have not been shown to be involved in the regulation of synaptic function. Therefore, we plan to explore the molecular mechanism underlying the regulation of synaptic function by circRNAs in the progression of POD in further experiments.

### Limitations

First, the assessment of cognitive impairment mainly relied on the TFC test and did not use the Morris water maze, but HE staining of hippocampal neurons provided new evidence. Second, the possible role of circRNA is mainly based on bioinformatics prediction. Although qRT–PCR verified the differential gene expression level, whether the identified circRNA truly regulates the progression of POD still needs further experimental verification. Third, we only analyzed circRNAs associated with POD in tibial fractures and did not verify whether other types of surgery, such as splenectomy, had similar results. Fourth, our short follow-up time made it impossible to evaluate the influence of circRNAs on long-term cognitive impairment.

## Conclusion

We screened the differentially expressed circRNAs in the hippocampus of POD mice using gene chip technology and predicted the genes with significantly differentially expressed circRNAs and related pathways through GO functional enrichment and KEGG pathway analysis. Combined with literature reports and expanded sample validation, we screened some interesting circRNA and parental gene pairs. It is hypothesized that these circRNAs may interact with their corresponding encoding genes to regulate the function of target genes. However, whether these associations play an important role in POD development remains to be further studied.

## Data Availability Statement

The original contributions presented in the study are publicly available. This data can be found here: https://www.ncbi.nlm.nih.gov/geo/query/acc.cgi?acc=GSE190880.

## Ethics Statement

This animal study was reviewed and approved by Ethics Committee of the First Affiliated Hospital of Chongqing Medical University (2021-718).

## Author Contributions

WR conceived and designed the study and interpreted the results. NL, RY, and ZW performed the experiments and prepared the initial draft of the manuscript. NL and JG supervised the project. All authors read and approved the final submission.

## Conflict of Interest

The authors declare that the research was conducted in the absence of any commercial or financial relationships that could be construed as a potential conflict of interest.

## Publisher’s Note

All claims expressed in this article are solely those of the authors and do not necessarily represent those of their affiliated organizations, or those of the publisher, the editors and the reviewers. Any product that may be evaluated in this article, or claim that may be made by its manufacturer, is not guaranteed or endorsed by the publisher.
